# Provincial Differences and Dynamic Changes in Mariculture Efficiency in China: Based on Super-SBM Model and Global Malmquist Index

**DOI:** 10.3390/biology9010018

**Published:** 2020-01-16

**Authors:** Xuan Yu, Qiuguang Hu, Manhong Shen

**Affiliations:** 1 Business School, Ningbo University, Ningbo 315211, China; huqiuguang@nbu.edu.cn; 2 Donghai Institute, Ningbo University, Ningbo 315211, China

**Keywords:** mariculture efficiency, China, super-SBM, GMI

## Abstract

The continued growth in mariculture efficiency in China is vital in ensuring that the country will continue to be the primary source of its own food in the 21st century. A large gap exists between the input and output of mariculture in China’s coastal provinces. Hence, methods to improve mariculture efficiency can be developed by analyzing provincial differences and dynamic changes in mariculture efficiency. These methods are important in solving China’s food security problems. This study uses the super-slacks-based measure model and global Malmquist index to measure the mariculture efficiency scores and their changes in China’s nine coastal provinces from 2004 to 2016. Results suggest that the mariculture efficiencies in Guangxi, Hainan, Fujian, Shandong, and Guangdong are higher than those in Jiangsu, Zhejiang, Liaoning, and Hebei. The mariculture efficiency in China increased by 6.45% from 2004 to 2016, and technological progress was the main driving force for this. The authors combine the results with the mariculture characteristics in each province and present the following findings: (1) mariculture scale affects mariculture efficiency; (2) efficient mariculture relies on a good marine ecosystem; (3) policy adjustments significantly affect the development of mariculture; (4) the key to improving mariculture efficiency is enhancing mariculture technology.

## 1. Introduction

China is the most populous country in the world; its population is 1.39 billion and will reach 1.44 billion in 2030, according to United Nations Population Division forecasts [1,2]. On the one hand, the national diet structure has been upgraded with income growth, and meat consumption continues to increase. On the other hand, the potential for food production increase from land areas is limited because of limited natural resources, such as cultivated land and freshwater. Hence, an important question should be answered: who will feed China? This shortage is an important issue that the Chinese government must face [3,4,5].

Given the severe food security situation, developing the food supply function of the ocean has become an important means of ensuring food supply [6,7,8]. Fish provides essential proteins and amino acids for humans [9]. From 2000 to 2016, global aquaculture grew at an average annual rate of 5.8%, which was higher than those of other major production sectors. Between 1961 and 2016, the average annual growth rate of the global consumption of fish food (3.2%) exceeded the population growth rate (1.6%), which was also higher than the growth rate of all terrestrial animal meat consumption (2.8%). The importance of fish in food supply is increasingly pronounced in developing countries and countries experiencing food shortages [10,11]. 

To date, China is a major producer of farmed food fish and has produced more of such food items than the rest of the world combined every year since 1991 [10]. Before the reform and opening up, China’s fish acquisition has relied mainly on marine fishing. However, the increasing fishing intensity has caused an excessive consumption of marine fishery resources, and fish can no longer be caught in some areas [12,13]. Coupled with advances in marine aquaculture technology, the supply structure of fish has shifted to mainly mariculture since 1986. Recent statistics have shown that China’s mariculture production accounted for 60% of total seawater production in 2017; hence, China has become the world’s largest mariculture country [10,14].

The following question should now be answered: to what extent can mariculture solve China’s food security problems in the future? The answer to this question generally depends on mariculture efficiency. Investment in production factors, such as mariculture area, labor, and fishing vessels, will not increase significantly or may even decrease in the future, owing to the deteriorating ecological environment in coastal waters and the continuous upgrading of the marine industry structure. Therefore, improving the mariculture efficiency and increasing the production of seafood per unit area have become key factors in the development of mariculture. China’s marine aquaculture administrative system has a central-to-local structure. That is, the central government is responsible for formulating the overall plan, whereas the provinces create specific implementation plans on the basis of the actual conditions of the regions [15]. However, this management system has caused gaps in the input and output of mariculture in the provinces, which has caused differences in mariculture efficiency. Hence, we can propose methods to improve mariculture efficiency by assessing the differences and changes in mariculture in each province, combining them with the actual conditions of mariculture in each province, and finding the cause of these differences.

Many researchers have given some research results on the efficiency of China’s marine fishery. For example, Chen (2017) measured and decomposed the marine fishery productivity efficiency by using the Global Malmquist productivity index, the results confirmed that significant differences exist between different regions of China’s marine fishery TFP (total factor productivity) [16]. Han (2019) analyzed the temporal and spatial evolution characteristics of China’s marine fishery ecological efficiency from 2006 to 2015, the results showed that the average value of marine fishery eco-efficiency in China was at a moderate level and fluctuating downward, while the regional differences showed a trend of fluctuation and expansion [17]. Sun (2017) evaluated the economic efficiency of marine fishery in 11 coastal provincial-level areas of China from 2004 to 2015 by using SBM (slacks-based measure) model, the results suggested that the economic efficiency of marine fishery remains low, which indicating that the marine fishery economic restructuring and upgrading has not yet achieved the desirable effect [18]. 

However, there are still not many studies on the mariculture efficiency in China. Previous studies have argued the ecological efficiency and technical efficiency of mariculture in China, such as Qin (2018) and Gao (2018), but have not focused on the overall mariculture efficiency [19,20]. This study constructed an index system for assessing the mariculture efficiency from input and output perspectives. The super- slacks-based measure (super-SBM) model and global Malmquist index (GMI) were used to quantitatively analyze the provincial differences and dynamic changes in mariculture efficiency in China. Thereafter, the authors combined the results with the actual mariculture situation in each province. Four aspects of the causes were analyzed: mariculture technology, mariculture scale, marine environment, and policy changes. We present the key points that China should focus on to improve the mariculture efficiency in the future and provide support to solve food security problems.

## 2. Materials and Methods

### 2.1. Super-SBM

This study uses the super-SBM model to measure the static score of mariculture efficiency. This model is a type of data envelopment analysis (DEA). The majority of the DEA models can evaluate efficiency only from a single input-oriented or output-oriented approach and cannot simultaneously consider input reductions and output increases. This limitation causes a deviation between the actual and theoretical values of efficiency. Tone (2001) proposed an SBM of efficiency, which is non-radial and deals with input/output slacks directly. This model differs from traditional radial measures of efficiency, which do not consider the existence of slacks, and can realistically reflect the actual situation of each production factor and accurately measure the efficiency of each decision-making unit (DMU) [21]. However, multiple DMUs that have their full efficient status denoted by unity (or 100%) cannot be compared. To discriminate between these efficient DMUs, Tone (2002) proposed an SBM of super-efficiency, which can rank these efficient DMUs.

If the presence of n DMUs is assumed, then $x\in R^{m}$ and $y\in R^{q}$ are input and output factors of mariculture, respectively. We define matrix $X=\left[x_{1},x_{2},\cdots ,x_{n}\right]\in R^{m\times n}$, *Y*$=\left[y_{1},y_{2},\cdots ,y_{n}\right]\in R^{q\times n}$, and the dataset is positive. That is, X>0, Y>0. The production possibility set *P* is defined as:

(1) P={(x,y)|x≥Xλ, y≤Yλ,λ≥0}

where λ is a non-negative vector in $R^{n}$. We consider an expression for describing a certain DMU $\left(x_{0},y_{0}\right)$ as:

(2) $$x_{0}=X\lambda +s^{-}$$

(3) $$y_{0}=Y\lambda -s^{+}$$

where λ≥0, $\;s^{-}\geq 0$ and $s^{+}\geq 0$. The vectors $s^{-}\in R^{m}$ and $s^{+}\in R^{q}$ indicate the input excess and output shortfall of this expression, respectively, and are called slacks [22]. Using slacks, we define the SBM model:

(4) $$\rho =min\frac{1-\frac{1}{m}\sum_{i=1}^{m}s_{i}^{-}/x_{i0}}{1+\frac{1}{q}\sum_{i=1}^{q}s_{i}^{+}/y_{i0}}\;s.t.\;\;x_{0}=X\lambda +s^{-},\;y_{0}=Y\lambda -s^{+}\;\lambda \geq 0,S^{-}\geq 0,S^{+}\geq 0$$

We continue to define a production possibility set $P\backslash \left(x_{0},y_{0}\right)$, which exclude $\left(x_{0},y_{0}\right)$ from (X,Y). That is:

(5) $$P\backslash \left(x_{0},y_{0}\right)=\left\{\left(\bar{x},\bar{y}\right)|\bar{x}\geq \sum_{j=1}^{n}\lambda_{j}x_{j},\bar{y}\leq \sum_{j=1}^{n}\lambda_{j}y_{j},\bar{y}\geq 0,\lambda \geq 0\right\}$$

A subset $\bar{P}\backslash \left(x_{0},y_{0}\right)$ of $P\backslash \left(x_{0},y_{0}\right)$ can be defined as:

(6) $$\bar{P}\backslash \left(x_{0},y_{0}\right)=P\backslash \left(x_{0},y_{0}\right)\cap \left\{\bar{x}\geq x_{0}\;and\;\bar{y}\leq y_{0}\right\}$$

As a weighted $l_{1}$ distance from $\left(x_{0},y_{0}\right)$ to $\left(\bar{x},\bar{y}\right)\in \bar{P}\backslash \left(x_{0},y_{0}\right)$, the index θ as defined by

(7) $$\theta =\frac{\frac{1}{m}\sum_{i=1}^{m}\bar{x_{i}}/x_{i0}}{\frac{1}{q}\sum_{r=1}^{q}\bar{y_{r}}/y_{r0}}\;$$

According to the interpretation of Tone (2002), θ is a product of the distance in the input space and output space, both indices are dimensionless. The super-SBM model can be defined as follows:

$$\theta^{*}=min\frac{\frac{1}{m}\sum_{i=1}^{m}\bar{x_{i}}/x_{i0}}{\frac{1}{q}\sum_{r=1}^{q}\bar{y_{r}}/y_{r0}}$$

$$s.t.\;\;x_{0}=X\lambda +s^{-},\;y_{0}=Y\lambda -s^{+}\;$$

$$\;\bar{x}\geq \sum_{j=1}^{n}\lambda_{j}x_{j}$$

$$\bar{y}\leq \sum_{j=1}^{n}\lambda_{j}y_{j}$$

$$\sum_{j=1}^{n}\lambda_{j}=1$$

(8) $$\bar{x}\geq x_{0},\bar{y}\leq y_{0},\bar{y}\geq 0,\bar{\lambda }\geq 0,S^{-}\geq 0,S^{+}\geq 0\;$$

In formula 1, $\theta^{*}$ is the efficiency score, *x* and *y* represent input and output respectively, *m* and *q* are the number of input and output indicators, $S^{-}$ and $S^{+}$ are slacks of input and output, meanwhile, λ is the weight vector. We define that DMU is SBM-efficient if *θ* ≥ 1 and DMU is SBM-inefficient if *θ* < 1. In this article, the authors use DEA-solver and MaxDEA Ultra to calculate super efficiency score of mariculture.

### 2.2. GMI

The mariculture efficiency score, measured using super-SBM, cannot simultaneously compare the two dimensions of time and space. Hence, this study uses GMI to reflect the dynamic changes in mariculture efficiency. Consider a panel of I DMUs and T time periods. DMUs use input x to produce output y. A global benchmark technology is defined as $T_{c}^{G}=\mathrm{conv}\;\left\{T_{c}^{1}\cup \ldots \cup T_{c}^{T}\right\}$. The subscript “c” indicates that the global benchmark technologies satisfy constant returns to scale.

A GMI is defined on $T_{c}^{G}$ as follows:

(9) $$M_{c}^{G}\left(x^{t},y^{t},x^{t+1},y^{t+1}\right)=\frac{D_{c}^{G}\left(x^{t+1},y^{t+1}\right)}{D_{c}^{G}\left(x^{t},y^{t}\right)}\;$$

Both indexes compare $\left(x^{t+1},y^{t+1}\right)$ with $\left(x^{t},y^{t}\right)$, but they use different benchmarks. Given only one global benchmark technology, the geometric mean convention is not needed when defining the global index.

Where the output distance functions

(10) $$D_{c}^{G}\left(x,y\right)=min\left\{\phi >0|\left(x,y/\phi \right)\in T_{c}^{G}\right\}$$

$M_{c}^{G}$ is decomposed as follows:

(11) $$\begin{array}{ll} M_{c}^{G}\left(x^{t},y^{t},x^{t+1},y^{t+1}\right) & =\frac{D_{c}^{t+1}\left(x^{t+1},y^{t+1}\right)}{D_{c}^{t}\left(x^{t},y^{t}\right)}\times \left\{\frac{D_{c}^{G}\left(x^{t+1},y^{t+1}\right)}{D_{c}^{t+1}\left(x^{t+1},y^{t+1}\right)}\times \frac{D_{c}^{t}\left(x^{t},y^{t}\right)}{D_{c}^{G}\left(x^{t},y^{t}\right)}\right\}\\ & =\frac{TE_{c}^{t+1}\left(x^{t+1},y^{t+1}\right)}{TE_{c}^{t}\left(x^{t},y^{t}\right)}\times \left\{\frac{D_{c}^{G}\left(x^{t+1},y^{t+1}\right)/D_{c}^{t+1}\left(x^{t+1},y^{t+1}\right)}{D_{c}^{G}\left(x^{t},y^{t}\right)/D_{c}^{t}\left(x^{t},y^{t}\right)}\right\}\\ & =EC_{c}\times \left\{\frac{BPG_{c}^{G,t+1}\left(x^{t+1},y^{t+1}\right)}{BPG_{c}^{G,t}\left(x^{t},y^{t}\right)}\right\}=EC_{c}\times BPC_{c}\; \end{array}$$

where $EC_{c}$ is the technical efficiency change index and $BPC_{c}$ is the technical change index. Compared with the contemporaneous Malmquist index, the GMI and each of its components are circular. Moreover, GMI provides single measures of productivity change and its components, and it is immune to LP (linear programming) infeasibility [23]. In this article, the authors use MaxDEA Ultra to calculate GMI of mariculture efficiency.

### 2.3. Evaluation Indicator System of Mariculture Efficiency

The use of the super-SBM model and GMI relies on the effective input–output indicators of mariculture productivity. Table 1 shows the evaluation indicator system of mariculture efficiency. In terms of input indicators, Land, labor, and capital are the most basic factors of production according to economics. Thus, the authors select the mariculture area, number of mariculture workers as the land and labor factors. The most suitable indicator of capital is the investment in fixed assets of mariculture, but China’s official statistics do not include this data. Considering the availability of data and refer to previous studies [4,17,18,19,20,24], the mariculture fishing vessels was chosen to reflect capital. The output indicators of mariculture mainly include two aspects: yield and production value [16,18]. Considering the large differences in the value of unit weight of different aquatic products, the authors selected mariculture yield and mariculture production value as the output indicators of mariculture. All data are from the China Fishery Statistical Yearbook.

### 2.4. Study Area

**Figure 1:**
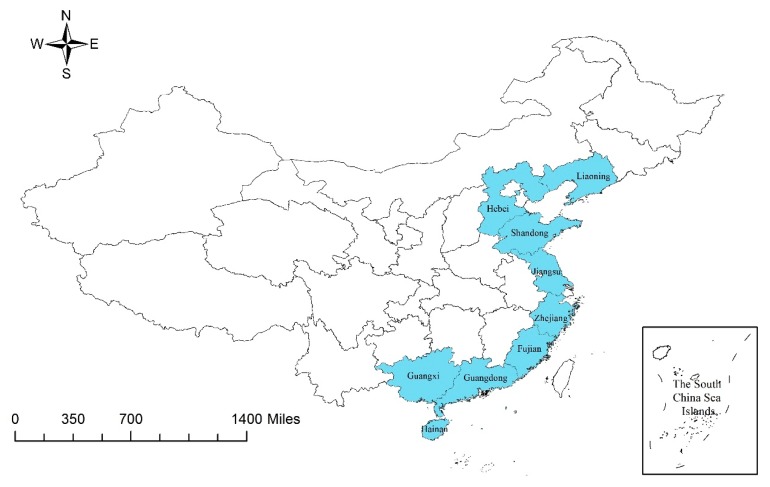
Location of China’s nine coastal provinces.

This study uses the super-SBM model and GMI to examine the regional differences and dynamic changes in mariculture efficiency in China’s coastal regions. The coastal area of China spans nine provinces, as follows (from north to south): Liaoning, Hebei, Shandong, Jiangsu, Zhejiang, Fujian, Guangdong, Guangxi, and Hainan (Figure 1). This research excludes Tianjin and Shanghai because they are direct-controlled municipalities and their mariculture scale is not comparable with those of the nine provinces. Moreover, the current study does not include Hong Kong, Macao, and Taiwan because of lack of data.

**Figure 2:**
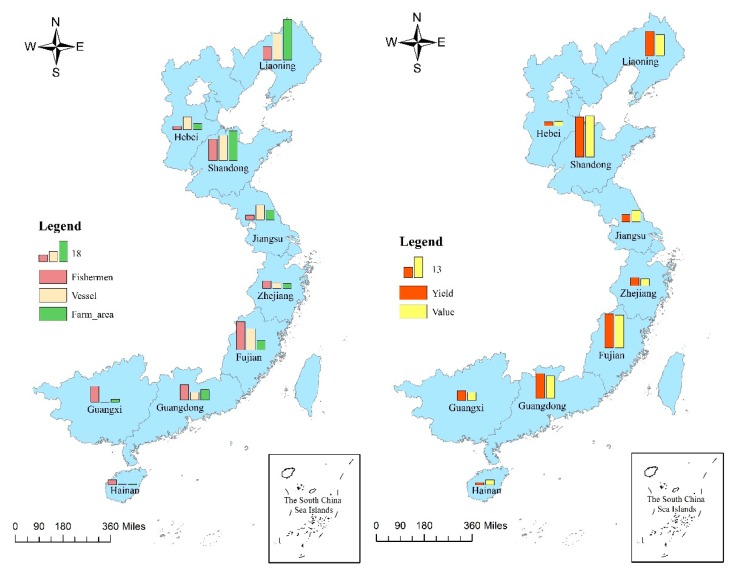
Input and output gaps in mariculture in China’s nine coastal provinces, 2016.

Figure 2 shows the input and output gaps in mariculture in China’s nine coastal provinces in 2016. The percentage data in the figure represent the proportion of each indicator to the total. Shandong, Fujian, and Liaoning are far superior to the remaining provinces in terms of marine aquaculture investment. Shandong’s labor input, fishing vessel input, and aquaculture area are sizably large. Fujian’s labor and fishing vessel inputs are significantly higher than those in the farming area. By contrast, the scale of marine aquaculture in Liaoning is mainly reflected by its farming area and fishing vessels. In terms of mariculture output (yield or value), Shandong, Fujian, Liaoning, and Guangdong are larger than Guangxi, Zhejiang, Jiangsu, Hebei, and Hainan. Shandong ranks first, whereas Hainan ranks last.

Table 2 lists the yield and scale of the four major mariculture species (fish, shrimp crab and shellfish) in China’s nine coastal provinces in 2018. In terms of fish and crab production, Guangdong and Fujian together account for more than half of China. In shrimp, the supply is mainly from Guangdong and Guangxi. But in terms of shellfish, Shandong and Fujian are the major breeding provinces. The above results show that there is not only a significant gap in the scale of mariculture in the coastal provinces of China, but also the main mariculture products are significantly different.

## 3. Results

The authors added each indicator of the nine provinces and used the DEA solver to measure the efficiency score of China’s mariculture in 2004–2016. The results shown in Table 3 suggest that China’s mariculture efficiency experienced a “U”-like trend in 2004–2016 (i.e., from SBM-efficient to SBM-inefficient to SBM-efficient).

Thereafter, the authors calculated the efficiency scores on the basis of the super-SBM model and GMI by using the software MaxDEA, which is provided by the Beijing Realworld Software Company (Beijing, China), to analyze the differences and changes in marine aquaculture efficiency across the provinces.

Table 4 displays the mariculture efficiency scores and their ranking in the nine provinces of China’s coastal areas from 2004 to 2016. Given the limitation of this paper, only the results from 2004, 2008, 2012, and 2016 are shown in the text. The complete results can be obtained from Appendix A. The calculation results indicate that we can divide the nine provinces into two types.

Type 1: Guangxi, Hainan, Fujian, Shandong, and Guangdong. The mariculture efficiency in these provinces is high. Guangdong was SBM-inefficient for a few years, but the mariculture efficiency in the four other provinces was SBM-efficient annually.

Type 2: Jiangsu, Zhejiang, Liaoning, and Hebei. The mariculture efficiency in these provinces is low. The mariculture efficiency was SBM-inefficient in the majority of years, and Hebei remains SBM-inefficient.

**Figure 3:**
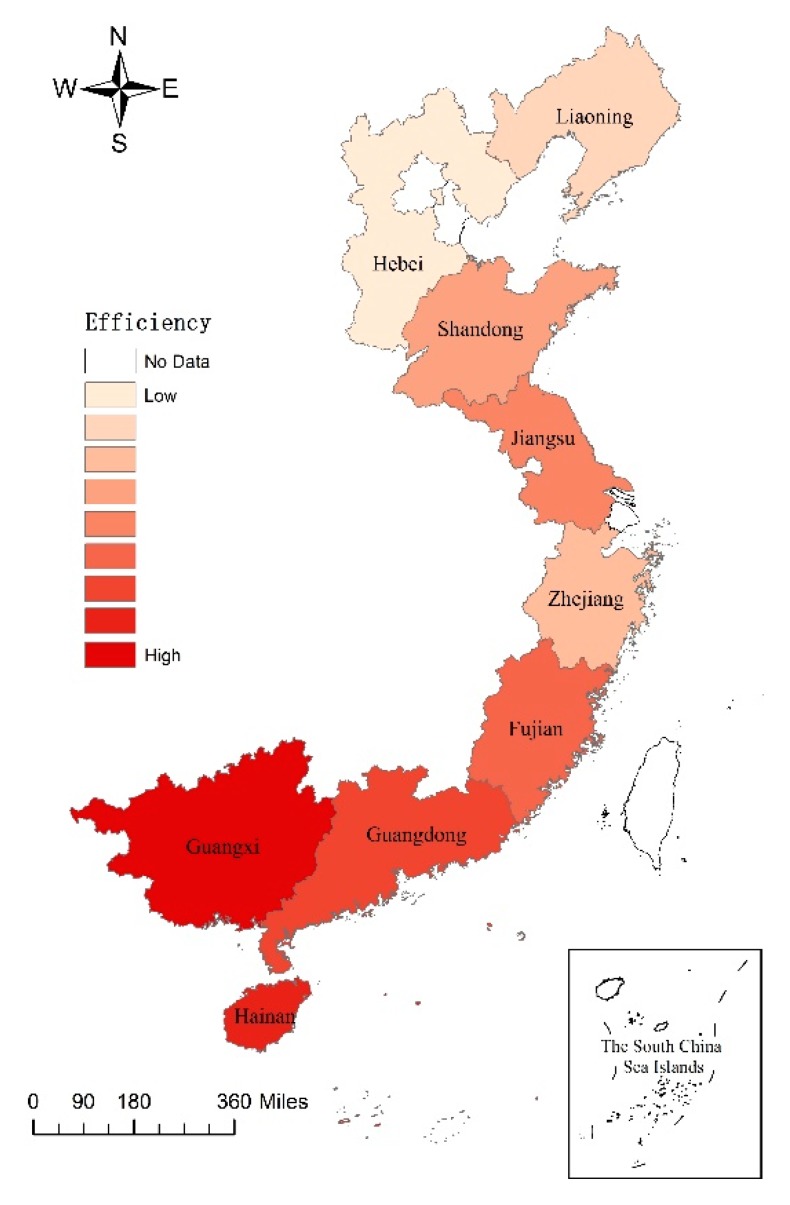
Difference in spatial distribution of the marine aquaculture efficiency in China, 2016.

Figure 3 shows the difference in the spatial distribution of marine aquaculture efficiency in China in 2016. The distribution pattern of marine aquaculture efficiency in China is approximately “south high and north low”.

### Dynamic Changes in Mariculture Efficiency

Table 5 shows the changes in mariculture efficiency in the nine provinces and the total of China’s coastal areas from 2004 to 2016. The results show that the overall GMI is 1.0645, thereby indicating that the mariculture efficiency in China increased by 6.45% in 2004–2016. The decomposition results of GMI show that ECI and BPCI are 0.9899 and 1.0754, which suggest that the main driver of mariculture efficiency growth is technological advances in mariculture. Similar to the overall level, the mariculture GMI in each province shows a growth trend, and the main motivation is technological progress.

**Figure 4:**
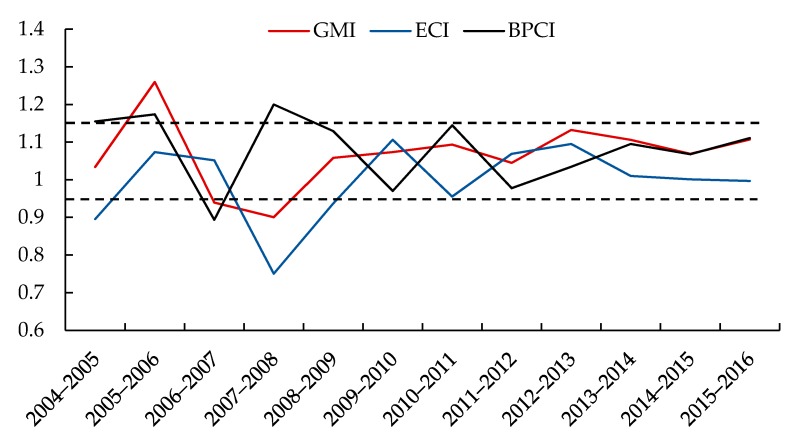
Change rate in the marine aquaculture efficiency in China, 2004–2016.

In accordance with the calculation results, a line diagram is used to describe the changes in mariculture’s GMI in China from 2004 to 2016. Figure 4 reveals that the period of intense fluctuation is mainly concentrated during the 11th Five-Year Plan (2006–2010). After 2010, the indices are primarily concentrated between 0.95 and 1.15.

## 4. Discussion

Provinces in China generally differ in terms of mariculture efficiency. Guangxi, Hainan, Fujian, Shandong, and Guangdong have high mariculture efficiency, whereas Jiangsu, Zhejiang, Liaoning, and Hebei have low mariculture efficiency. The mariculture efficiency in China increased by 6.45% in 2004–2016, and technological progress is the main driving force for this improvement. The authors combine these results with the mariculture characteristics in each province, and explain the reasons for the differences of mariculture efficiency from four aspects: mariculture technology, mariculture scale, marine environment, and policy changes. The findings are discussed as follows:

(1) Mariculture scale affects aquaculture efficiency. The authors combine Figure 2 with Table 4, and the mariculture standards are consistently ranked among the top two provinces in Guangdong and Fujian. Mariculture efficiency has constantly been SBM-efficient, whereas the mariculture efficiency of Hebei and Zhejiang, which have a smaller scale mariculture, is SBM-inefficient. With the expansion of mariculture, scale effects can be produced within a certain range, which is beneficial to the optimization of labor and capital allocation and will reduce production costs. Producers with scale advantages can improve their mechanization level, promote the application of new farming techniques, and increase the efficiency of mariculture by the scale effect caused by overall improvements in farming level. In addition, large-scale mariculture areas can effectively avoid breeding risks and have the power to cope with the impact of emergencies on mariculture;
(2) Efficient mariculture relies on a good marine ecosystem. The mariculture efficiency in Guangxi and Hainan is SBM-efficient annually, and the seawater quality in the coastal waters is “excellent” throughout the year. By contrast, seawater quality is low in most areas of the East China Sea, such as Zhejiang and Jiangsu. Thus, the mariculture efficiency in these provinces is low. Moreover, the harsh marine ecological environment prevents the mariculture in Liaoning from playing its due scale effect, and a large amount of marine aquaculture investment is indirectly proportional to its output. Mariculture requires seawater as carrier. A good marine ecological environment can provide high-quality habitats for aquaculture organisms. Consequently, the survival rate of cultured organisms is high, growth is fast, disease is reduced, and yield per unit area is increased. Furthermore, a good marine ecological environment can increase the natural bait in seawater and reduce the use of artificial bait, thereby decreasing the cost of mariculture, making the cultured meat type resemble the wild type, and enhancing the value of aquatic products;
(3) Policy adjustments significantly affect the development of mariculture. The volatility of China’s mariculture GMI and its decomposition index is relatively evident during the 11th Five-Year Plan period, which is the period of change in the growth pattern of China’s aquaculture industry. The National Fisheries Development 11th Five-Year Plan proposes to “promote the transformation of aquaculture growth mode.” Subsequently, the Ministry of Agriculture of the People’s Republic of China promulgated the “Implementation Plan for the Transformation of Growth Patterns in Aquaculture Industry” to “advocate and promote the healthy farming methods of aquaculture” to realize “the transformation of the aquaculture industry from the pursuit of quantity to quantity and quality, efficiency and ecological growth mode.” The Ministry of Agriculture has successively issued various supporting rules and regulations to promote the implementation of the policy. Along with the continuous adjustment of mariculture policies, the rate of change in mariculture efficiency also constantly fluctuates. When policies are on track, the changes in mariculture efficiency gradually stabilize;
(4) The key to improving mariculture efficiency is to improve mariculture technology. Development economics state that the level of technology directly affects production efficiency. The decomposition results of GMI indicate that the driving force for the improvement in mariculture efficiency in China is mainly the advancement of mariculture technology. Advances in mariculture technology will increase mariculture efficiency in the following ways. First, advances in mariculture technology will improve mariculture tools, greatly increase the output of aquatic products, and promote the continuous expansion of the types of aquatic products that can be cultured. Second, advances in mariculture technology will promote the quality of mariculture labor. On the one hand, advanced mariculture techniques require that mariculture personnel should have be of a high quality and mariculture personnel should receive additional education and training. On the other hand, the modernization of mariculture technology enables workers to improve their skills in specialized labor. Moreover, advances in mariculture technology reduce labor time and provide the needed time conditions for mariculture personnel to improve their education and overall quality. Third, advances in mariculture technology enhance the management efficiency of mariculture. The use of information technology provides a convenient communication tool for mariculture management. Standardized production methods and digital operation methods greatly decrease management costs. Thus, management capabilities and management levels can be continuously updated.

These discussions have several policy implications for improving mariculture efficiency. First, the scale effect should be applied to promote the improvement of mariculture efficiency for provinces with large-scale mariculture. Second, the issue of marine environmental capacity should be considered during mariculture, the marine ecological environment must be protected, and the healthy and sustainable development of mariculture should be promoted. Third, the central and local governments should provide a reasonable mariculture policy to indicate the direction of mariculture development. Lastly, the government should increase funding and talent investment in mariculture technology, innovate mariculture technology, and support the development of mariculture with high-level mariculture technology.

## 5. Conclusions

This study uses the super-slacks-based measure model and global Malmquist index to measure the mariculture efficiency scores and their changes in China’s nine coastal provinces from 2004 to 2016. Results suggest that the mariculture efficiencies in Guangxi, Hainan, Fujian, Shandong, and Guangdong are higher than those in Jiangsu, Zhejiang, Liaoning, and Hebei. The mariculture efficiency in China increased by 6.45% from 2004 to 2016, and technological progress was the main driving force for this. The authors discussed the reasons for the differences of mariculture efficiency from four aspects: mariculture technology, mariculture scale, marine environment, and policy changes. Based on the results, the authors put forward several policy suggestions for improving mariculture efficiency in China in the future.

## Figures and Tables

**Table 1:** Evaluation indicator system of mariculture efficiency.

Components	Variables	References
Input	Mariculture area (hm^2^)	[4,16,17,18,19,20,24]
	Mariculture workers (person)	
	Mariculture fishing vessels (t)	
Output	Mariculture yield (t)	
	Mariculture production value (10,000 yuan)	

**Table 2:** The yield and scale of the major mariculture species in China’s nine coastal provinces, 2018.

Province	Fish		Shrimp		Crab		Shellfish	
	Yield (t)	Scale (%)	Yield (t)	Scale (%)	Yield (t)	Scale (%)	Yield (t)	Scale (%)
Heibei	11,648	0.78	29,531	2.10	1549	0.53	433,107	3.00
Liaoning	71,841	4.81	34,678	2.46	6285	2.14	2,294,524	15.89
Jiangsu	83,925	5.61	80,492	5.71	34,797	11.84	667,450	4.62
Zhejiang	44,692	2.99	62,717	4.45	49,573	16.87	959,930	6.65
Fujian	391,007	26.15	128,610	9.13	73,467	25.01	3,028,196	20.97
Shandong	111,454	7.45	147,360	10.46	19,679	6.70	4,148,921	28.73
Guangdong	594,793	39.78	507,543	36.02	74,578	25.38	1,898,078	13.15
Guangxi	59,748	4.00	296,528	21.04	19,521	6.64	982,804	6.81
Hainan	124,554	8.33	115,429	8.19	14,348	4.88	26,292	0.18

**Table 3:** Mariculture efficiency scores of China, 2004–2016.

Year	Score	Status
2004	1.0030	SBM-efficient
2005	1.0066	SBM-efficient
2006	1.0956	SBM-efficient
2007	0.7158	SBM-inefficient
2008	0.5879	SBM-inefficient
2009	0.5896	SBM-inefficient
2010	0.6325	SBM-inefficient
2011	0.7121	SBM-inefficient
2012	0.7598	SBM-inefficient
2013	0.8233	SBM-inefficient
2014	0.8771	SBM-inefficient
2015	1.0101	SBM-efficient
2016	1.0667	SBM-efficient

**Table 4:** Mariculture efficiency in the coastal areas of China, 2004–2016.

Province	2004		2008		2012		2016	
	Score	Rank	Score	Rank	Score	Rank	Score	Rank
Liaoning	1.01	7	0.45	8	0.49	8	0.58	8
Hebei	0.55	9	0.28	9	0.37	9	0.43	9
Shandong	1.04	6	1.06	4	1.15	5	1.06	6
Jiangsu	1.11	4	0.64	6	0.55	7	1.09	5
Zhejiang	1.06	5	0.47	7	0.62	6	0.6	7
Fujian	1.24	3	1.07	3	1.2	4	1.16	4
Guangdong	0.82	8	1.02	5	1.36	2	1.27	3
Guangxi	1.94	1	1.37	1	3.76	1	3.58	1
Hainan	1.6	2	1.34	2	1.29	3	1.3	2

**Table 5:** Geometric mean of the change rate in the marine aquaculture efficiency in China, 2004–2016.

Province	GMI	ECI	BPCI
Liaoning	1.0605	0.9562	1.1090
Hebei	1.0534	0.9799	1.0750
Shandong	1.0834	1.0000	1.0834
Jiangsu	1.1177	1.0000	1.1177
Zhejiang	1.0385	0.9582	1.0839
Fujian	1.0714	1.0000	1.0714
Guangdong	1.0927	1.0169	1.0745
Guangxi	1.0000	1.0000	1.0000
Hainan	1.0675	1.0000	1.0675
Overall	1.0645	0.9899	1.0754

## Appendix A.

**Table A1:** Mariculture efficiency in coastal areas of China, 2004–2016.

Year	Type	Liaoning	Hebei	Shandong	Jiangsu	Zhejiang	Fujian	Guangdong	Guangxi	Hainan
2004	score	1.01	0.55	1.04	1.11	1.06	1.24	0.82	1.94	1.6
	rank	7	9	6	4	5	3	8	1	2
2005	score	0.62	0.39	1.07	1.09	0.68	1.17	1.1	4.69	1.24
	rank	8	9	6	5	7	3	4	1	2
2006	score	0.76	0.41	1.09	1.01	1.05	1.12	1.04	1.12	2.91
	rank	8	9	4	7	5	2	6	2	1
2007	score	1.02	0.49	1.09	1.02	1.05	1.17	1.1	1.06	2.83
	rank	7	9	4	7	6	2	3	5	1
2008	score	0.45	0.28	1.06	0.64	0.47	1.07	1.02	1.37	1.34
	rank	8	9	4	6	7	3	5	1	2
2009	score	0.42	0.26	1.1	0.65	0.45	1.02	0.65	1.48	1.31
	rank	8	9	3	5	7	4	5	1	2
2010	score	0.5	0.36	1.06	0.59	0.48	1.11	1	3.95	1.31
	rank	7	9	4	6	8	3	5	1	2
2011	score	0.42	0.28	1.28	0.45	0.65	1.2	1.25	4.4	1.32
	rank	8	9	3	7	6	5	4	1	2
2012	score	0.49	0.37	1.15	0.55	0.62	1.2	1.36	3.76	1.29
	rank	8	9	5	7	6	4	2	1	3
2013	score	0.56	0.41	1.16	1.03	0.62	1.18	1.14	3.81	1.31
	rank	8	9	4	6	7	3	5	1	2
2014	score	0.58	0.41	1.13	1.01	0.64	1.16	1.23	4.1	1.33
	rank	8	9	5	6	7	4	3	1	2
2015	score	0.55	0.43	1.13	1.01	0.65	1.16	1.11	4.2	1.27
	rank	8	9	4	6	7	3	5	1	2
2016	score	0.58	0.43	1.06	1.09	0.6	1.16	1.27	3.58	1.3
	rank	8	9	6	5	7	4	3	1	2

## References

[1] NBS (2018). China Statistical Yearbook.

[2] Department of Economic and Social Affairs (UN DESA) (2017). World population prospects, the 2017 Revision, Key Findings and Advance Tables.

[3] Fukase E., Martin W. (2014). Who Will Feed China in the 21st Century? Income Growth and Food Demand and Supply in China.

[4] Wang P.P., Ji J.Y. (2017). Research on China’s mariculture efficiency evaluation and influencing factors with undesirable outputs—An empirical analysis of China’s ten coastal regions. Aquac. Int..

[5] Han L.M., Li D.H. (2015). Blue Food System: Guarantee of China’s Food Security. Issues Agric. Econ..

[6] Pauly D., Watson R., Alder J. (2005). Global trends in world fisheries: Impacts on marine ecosystems and food security. Philos. Trans. R. Soc. B.

[7] Ricel J.C., Garcia S.M. (2011). Fisheries, food security, climate change, and biodiversity: Characteristics of the sector and perspectives on emerging issues. ICES J. Mar. Sci..

[8] Ronnback P., Bryceson I., Kautsky N. (2002). Coastal Aquaculture development in eastern Africa and the Western Indian Ocean: Prospects and problems for food security and local economies. AMBIO J. Hum. Environ..

[9] Merino G., Barange M., Blanchard J.L., Harle J., Holmes R., Allen I., Allison E.H., Badjeck M.C., Dulvy N.K., Holt J. (2012). Can marine fisheries and aquaculture meet fish demand from a growing human population in a changing climate?. Glob. Environ. Chang..

[10] FAO (2018). The State of World Fisheries and Aquaculture.

[11] Troell M., Naylor R.L., Metian M., Beveridge M., Tyedmers P.H., Folke C., Arrow K.J., Barrett S., Crepin A.S., Ehrlich P.R. (2014). Does aquaculture add resilience to the global food system?. Proc. Natl. Acad. Sci. USA.

[12] Shen Y.A., Wu H., Sun Z.N., Lin L.S. (2012). China’s marine fisheries: Achievements, problems and development approaches. Chin. Fish. Econ..

[13] Lu C.C., Zhao J.H. (2013). An investigation on the countermeasures against of “No fish in the East China Sea”. Chin. Fish. Econ..

[14] MARA (2018). China Fisheries Statistical Yearbook.

[15] Shen G.M., Heino M. (2014). An overview of marine fisheries management in China. Mar. Policy.

[16] Chen Z.L., Cheng Y.Y., Shen M.H. (2017). Study on marine fishery efficiency and regional differential in China. Sci. Technol. Econ..

[17] Han L.M., Ji X.Q., Hu Y., Cai X.Z. (2019). The spatial-temporal evolution of marine fishery eco-efficiency based on SBM model in China. Ocean Dev Manage.

[18] Sun K., Ji J.W., Li L.D., Zhang C., Liu J.F., Fu M. (2017). Marine fishery economic efficiency and its spatio-temporal differences based on undesirable outputs in China. Resour. Sci..

[19] Qin H., Zhang Y., Lu Y.Y. (2018). Measurement and analysis of China’s mariculture eco—economic efficiency: Based on SBM model. J. Agrotech. Econ..

[20] Gao J.J., Shi Q.H., Lu K. (2018). Study on technical efficiency evaluation of Chinese marine aquaculture. J. Agrotech. Econ..

[21] Tone K. (2001). A slacks-based measure of efficiency in data envelopment analysis. Eur. J. Oper. Res..

[22] Tone K. (2002). A slacks-based measure of super-efficiency in data envelopment analysis. Eur. J. Oper. Res..

[23] Pastor J.T., Lovell C.A.K. (2005). A global Malmquist productivity index. Econ. Lett..

[24] Yu S.H., Yu H.J. (2012). An empirical study on the coastal areas fishery efficiency in China: Based in DEA-Malmquist index. Chin. Fish. Econ..

